# Prevalence, drivers and surveillance of antibiotic resistance and antibiotic use in rural China: Interdisciplinary study

**DOI:** 10.1371/journal.pgph.0001232

**Published:** 2023-08-09

**Authors:** H. Lambert, X. Shen, J. Chai, J. Cheng, R. Feng, M. Chen, C. Cabral, I. Oliver, J. Shen, A. MacGowan, K. Bowker, M. Hickman, P. Kadetz, L. Zhao, Y. Pan, R. Kwiatkowska, X. Hu, D. Wang

**Affiliations:** 1 Bristol Medical School, University of Bristol, Bristol, United Kingdom; 2 School of Health Services Management, Anhui Medical University, Hefei, China; 3 Library Department of Literature Retrieval and Analysis, Anhui Medical University, Hefei, China; 4 Field Service, National Infection Service, UK Health Security Agency, Bristol, United Kingdom; 5 Fourth Affiliated Hospital of Anhui Medical University, Hefei, China; 6 Severn Pathology, North Bristol NHS Trust, Bristol, United Kingdom; 7 Institute for Global Health and Development, Queen Margaret University, Edinburgh, United Kingdom; 8 First Affiliated Hospital of Anhui Medical University, Hefei, China; 9 Anhui Provincial Hospital, Hefei, China; University of Oslo Faculty of Medicine: Universitetet i Oslo Det medisinske fakultet, NORWAY

## Abstract

This study aimed to characterise antibiotic prescribing and dispensing patterns in rural health facilities in China and determine the community prevalence of antibiotic resistance. We investigated patterns and drivers of antibiotic use for common respiratory and urinary tract infections (RTI/UTI) in community settings, examined relationships between presenting symptoms, clinical diagnosis and microbiological results in rural outpatient clinics, and assessed potential for using patient records to monitor antibiotic use. This interdisciplinary mixed methods study included: (i) Observations and exit interviews in eight village clinics and township health centres and 15 retail pharmacies; (ii) Urine, throat swab and sputum samples from patients to identify potential pathogens and test susceptibility; (iii) 103 semi-structured interviews with doctors, patients, pharmacy workers and antibiotic-purchasing customers; (iv) Assessment of completeness and accuracy of electronic patient records through comparison with observational data. 87.9% of 1123 recruited clinic patients were prescribed antibiotics (of which 35.5% contained antibiotic combinations and >40% were for intravenous administration), most of whom had RTIs. Antibiotic prescribing for RTIs was not associated with presence of bacterial pathogens but was correlated with longer duration of infection (OR = 3.33) and presence of sore throat (OR = 1.64). Fever strongly predicted prescription of intravenous antibiotics (OR = 2.87). Resistance rates in bacterial pathogens isolated were low compared with national data. 25.8% of patients reported antibiotics use prior to their clinic visit, but only 56.2% of clinic patients and 53% of pharmacy customers could confirm their prescription or purchase included antibiotics. Diagnostic uncertainty, financial incentives, understanding of antibiotics as anti-inflammatory and limited doctor-patient communication were identified as key drivers of antibiotic use. Completion and accuracy of electronic patient records were highly variable. Prevalence of antibiotic resistance in this rural population is relatively low despite high levels of antibiotic prescribing and self-medication. More systematic use of e-records and in-service training could improve antibiotic surveillance and stewardship in rural facilities. Combining qualitative and observational anthropological methods and concepts with microbiological and epidemiological investigation of antibiotic resistance at both research design and analytic synthesis stages substantially increases the validity of research findings and their utility in informing future intervention development.

## Introduction

Antimicrobial resistance (AMR), and especially resistance of bacterial pathogens to antibiotics (antibiotic resistance, ABR), is recognised as a major global health concern [1–3], with an estimated 4·95 million deaths globally associated with bacterial AMR in 2019 [4]. Bacterial antimicrobial resistance is partially driven by non-essential use of antibiotics in medical treatment;[5] and China is one of the world’s largest consumers of antibiotics, with prescription rates twice that recommended by the World Health Organisation, [6]. Global and regional comparison is difficult (China does not report data to the WHO Global Antimicrobial Resistance and Use Surveillance System, GLASS), [7] but there is substantial evidence that antibiotics are overused in healthcare [8, 9]. Rural areas have higher antibiotic prescribing rates than urban areas, [10] but government interventions to optimise antibiotic use have to date focused mainly on large (secondary and tertiary) city hospitals. Published evidence reporting high prevalences of antimicrobial resistance (AMR) and declining trends in antibiotic prescribing in China is based on national surveillance data (CARRS), point prevalence surveys and analysis of laboratory and prescribing data from tertiary hospital inpatients. Since 2015, an electronic record system has been operational in health care facilities in China, and information from this has provided valuable insights about patterns of antibiotic use. However little data from rural settings or lower level (first- and second-tier) health facilities are available and the reliability of secondary data sources such as electronic patient records, that are frequently used to evaluate the effects of policy changes, has not previously been investigated.

This paper presents findings from an interdisciplinary study to characterise antibiotic prescribing and dispensing patterns in rural health facilities of Anhui Province and measure the community prevalence of antibiotic resistance. Our aims were to identify key drivers of and patient pathways to antibiotic use (ABU) for common infections, assess relationships between presenting symptoms, clinical diagnosis and microbiological diagnosis in rural healthcare settings (which do not have laboratory facilities for culturing samples), and evaluate the potential for using patient records to monitor ABU. Such evidence is essential to support the development of effective interventions to promote antibiotic stewardship and reduce the burden of antibiotic resistance, as well as to identify accurate methods for monitoring changes in ABU and ABR over time. We chose to focus on outpatients presenting with urinary tract infections (UTI) and respiratory tract infections (RTI) as they comprise a high proportion of outpatient consultations for which antibiotics are used in general practice/primary (first-level) care, a setting which accounts for roughly three-quarters of antibiotics prescribed worldwide [11]. This paper, in synthesising findings from all study components (epidemiological, microbiological and social science), demonstrates the value of innovative cross-disciplinary research designs in which convergent methodological approaches provide insights beyond those which can be derived from exclusively epidemiological, microbiological, or social science research.

At the base of the medical system in rural China are village clinics (outpatient only) and township health centres (which have inpatient beds); [12] in urban areas equivalent services are provided by community health stations and community health centres. Like primary care institutions in European countries, they provide both preventive public health services and primary care. Hospitals across China are divided into three tiers; township and community health centres are categorised as primary tier hospitals, secondary tier hospitals include county, city and district hospitals, and tertiary tier hospitals with higher bed capacities offer specialist services and medical education at provincial or national level. As such, village clinics and community health stations—the lowest level health facilities in rural and urban areas respectively—are not part of China’s ‘three-tier’ medical system and are financially semi-autonomous, but they are overseen by Primary hospitals and provide many of the preventive services for which the latter are responsible.

Village clinics are staffed by village doctors who trained and are certified as Village Practitioners but do not have degree level medical qualifications, unlike the licensed doctors who work at township health centres and in higher tier health facilities [13]. Around two thirds of initial consultations occur in village (rural) or community (urban) clinics, and township or community health centres receive a slightly higher proportion (17%) than (urban) county-level hospitals (14%). Patients can seek treatment at any healthcare facility, but medical insurance systems incentivise use of first-tier care through greater reimbursement ratios. Almost all our study participants (99%) were members of the NACMIS (New Agricultural Cooperative Medical Insurance Scheme), a State-supported mutual health insurance scheme involving subsidised contributions, which covers hospitalisation and certain outpatient medical costs incurred at village clinics and township health centres [14]. Medicines including antibiotics can also be purchased with or without prescription at many retail pharmacies or ‘medicine shops’, although the government is increasingly restricting the (technically prohibited) sale of antibiotics that are not prescribed. The study was conducted in Anhui Province, which has a population of 62.6 million (roughly similar to that of the UK), of whom 46.7% reside in rural areas; the province’s social and cultural profile is considered representative of over 80% of the Chinese population, average life expectancy is 75.08 years and per capita GDP ranks 12th among China’s 23 provinces [15]. In common with much of rural China, the study sites have experienced substantial outmigration of younger adults to urban areas to find work, with poorer and less educated older people and ‘left-behind’ grandchildren overrepresented among rural residents [16].

## Methods

**Fig 1:**
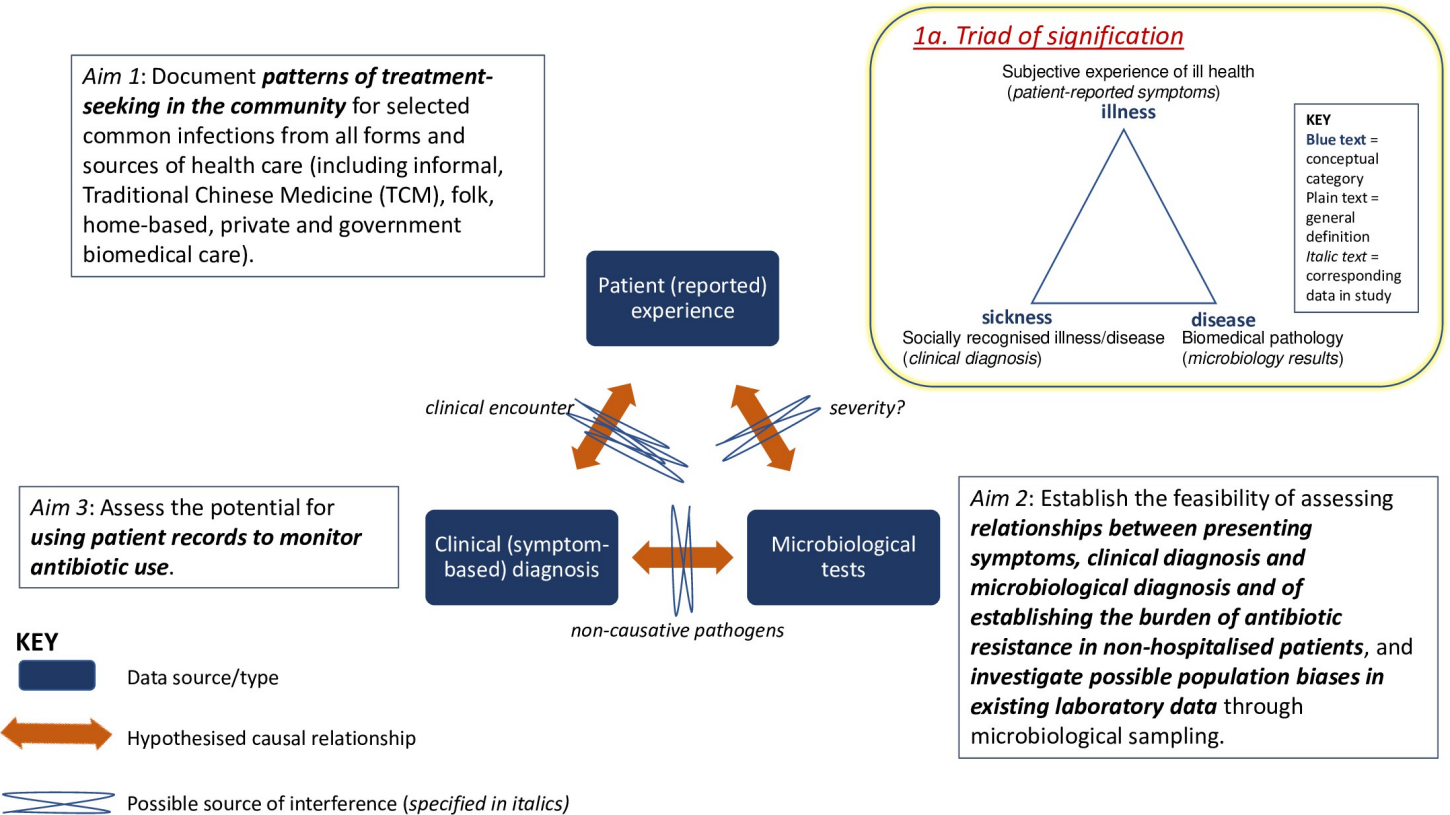
Research design and triangulation.

This interdisciplinary study comprised three main components: microbiological sampling and laboratory testing, clinical study and record review, and qualitative study and quantitative survey of antibiotic prescribing and purchasing practices. We used a convergent mixed-methods approach, [17] with data from linked studies initially analysed independently and findings synthesised at interpretation stage. The relationship between each study component was underpinned by a unifying conceptual framework which triangulates the three main research aims with their corresponding data sources (Fig 1) to achieve integration at design level [18]. This novel design draws on anthropological concepts that distinguish subjectively experienced ‘illness’ from biologically defined ‘disease’, [19–21] and from socially legitimated ‘sickness’, [22, 23] in a ‘triad of signification’ to capture the co-determining relationships between symptoms, the diseases of which they are symptomatic, and the diagnoses through which they are recognised and categorised. Each study component is summarised below; additional details of methods can be found in the published study protocol [24].

Data collection took place consecutively in four counties of Anhui Province, at one village clinic and one township health centre in each county. Health facilities were selected randomly from a list provided by the provincial health board of all potential facilities fulfilling set criteria (population size, location, transport links, patient footfall) in each county. Microbiological sampling and semi-structured observations were conducted in all eight health facilities, while exit surveys, interviews and observations in retail pharmacies were conducted in the first three study areas (six healthcare facilities and all local pharmacies, which varied from 4–8 in each study area). We also completed a complementary systematic scoping review to identify key influences on antibiotic use in China, the results of which are published separately [25].

### Ethics approval and consent to participate

The full research protocol was reviewed and approved by the Biomedical Ethics Committee of Anhui Medical University (reference number: 20170271). Participation of patients and doctors was voluntary and written informed consent was sought from all participants.

### Data access

Data referred to in this paper are available at the University of Bristol data repository, data.bris, at https://doi.org/10.5523/bris.1waogd70b72v2s329r0tajapp.

### Microbiological study

Eligible patients were recruited using consecutive sampling in each participating health facility. Inclusion criteria were ≥18 years, presenting at the recruitment site for their current illness for the first time during the study period, and diagnosis of exacerbation of COPD, upper RTI with cough, sore throat, or UTI (symptoms of frequent urination, urgency, pain/difficulty in urination, lower body itching (itchy urinary tract), ‘tummy dropping’ pain, turbid urination, incontinence, hematuria, or a diagnosis of urethritis). Specimen collection of sputum from RTI patients with productive cough, throat swabs from patients with sore throat and urine from patients with a UTI were performed by the attending doctor and placed into a sterilised tube for refrigeration and transportation to the participating laboratory within four hours according to a standard operating procedure. Vitek 2 compcat (BioMerieux, France) was used for drug susceptibility testing and results were interpreted according to CLSI reference guidelines [26]. Full details of bacterial identification and susceptibility testing are provided in the study protocol paper [24].

### Semi-structured direct observations

Following recruitment, participants were accompanied through their outpatient journey by a researcher who completed a recruitment proforma, clinic observation worksheet and exit survey. Presenting symptoms, tests ordered, diagnosis given and treatment prescribed were recorded through direct observation. At retail pharmacies or medicine shops, interactions between customers and pharmacy workers including symptoms presented, medicines and information requested/provided and medicines purchased were recorded over a two-week period in each shop.

### Structured exit surveys

All consenting patients recruited at health care facilities completed a researcher-administered questionnaire after their consultation to elicit details of symptoms, prior treatment, views on treatment and lay diagnoses. At retail pharmacies customers purchasing antibiotics were asked to complete a similar short structured exit survey on leaving the shop.

### In-depth interviews

A representative subsample of patients recruited at healthcare facilities were contacted for a follow-up interview at home, between one and two weeks after their clinic visit. A subsample of customers purchasing antibiotics over the counter were also interviewed. Semi-structured interviews were conducted with attending doctors at all participating health facilities at the end of the recruitment period and with workers at retail pharmacies.

### Patient record review

After study recruitment had been completed in each of the first four participating medical facilities, researchers revisited each facility to identify the electronic records (e-records) for the observed consultations. Researchers then compared data from clinic observations and recruitment proformas with the information in the e-records, using a bespoke template to document degree of agreement. Full methodological details of this study component are provided elsewhere [27].

### Analysis

Questionnaire responses were double-entered into a database using EPI DATA 3.1, then extracted and analysed using SPSS and Microsoft Excel 2013. Quantitative data analysis comprised descriptive estimations and multivariate logistic regression modelling to assess factors associated with antibiotic prescription from health care professionals and test relationships between observational data on clinical diagnoses and microbiological results. Initial coding of a subsample of interview transcripts in Mandarin and translated English versions was carried out by three interviewers and three UK-based researchers; codes were compared in Skype meetings to derive a dual-language coding framework for each qualitative dataset. Remaining transcripts were coded in Mandarin by interviewers using NVivo and analysed thematically.

## Results

We first provide a descriptive summary of our research activities by study setting and then present findings on patterns of antibiotic use drawing on clinic recruitment data and patient exit surveys, and on identification of bacterial pathogens and rates of antibiotic resistance from the microbiological study component. This is followed by statistical analysis of relationships between antibiotic prescribing, patient symptoms and microbiological results. Research findings from direct observations, exit interviews and in-depth interviews are then presented to shed light on reasons for the patterns of antibiotic use. The final section presents results from a comparison between directly observed clinical consultations and patient record data that has important implications for future monitoring and surveillance of antibiotic use.

### Study settings and activities

Table 1 summarizes study sites and main research activities by site. A total of 1123 eligible patients in the clinical settings were recruited into the microbiological study and completed a structured exit interview following their consultation. The microbiological study identified 153 bacterial isolates that were tested for sensitivity to 34 commonly used antibiotics. Separately, we observed 2383 encounters in retail pharmacies and conducted exit surveys with 364 customers who had bought antibiotics.

**Table 1:** Study settings and types of data collected.

*Data collection activities*	*Clinical*		*Community*	*Total*
	Township health centers	Village Clinics	Retail pharmacies	
Settings selected	4	4	15	23
Encounters observed (total)	668	455	729	1852
-symptomatic respiratory tract infections	641	427	720	1788
-symptomatic urine tract infections	27	28	9	64
Exit survey completed	668	455	364	1487
In-depth interviews (total)	49	30	24	103
-patients/customers	37	24	16	77
-doctors/shop keepers	12	6	8	26
Specimens collected and cultivated for bacteria	668	455	NA	1123
-sputum	440	243	NA	683
-throat swab	201	184	NA	385
-urine	27	28	NA	55
Bacterial isolates tested for antibiotic resistance (top 10 of sputum and throat swab samples)	240	112	NA	352
-*K*. *pneumonia*	63	22	NA	85
-*H*.*influenzae*	44	13	NA	57
-*H*.*parainfluenzae*	27	25	NA	52
-*P*.*aeruginosa*	17	5	NA	22
-*S*.*aureus*	14	4	NA	18
-*H*.*parahaemolyticus*	10	7	NA	17
-*M*.*catarrhalis*	8	4	NA	12
-*E*.*cloacae*	8	4	NA	12
-*K*.*aerogenes*	5	3	NA	8
-*S*.*pneumoniae*	4	4	NA	8

### Patterns of antibiotics use

84.3% to 93.2% of all recruited patients received a prescription containing antibiotics in the four project sites (87.8% of RTIs and 89.1% of UTIs); of these, between 45.5% and 41.1% of prescriptions were for intravenous (IV) antibiotics (see Table 2). Patients presenting at township health centres were more likely to receive IV antibiotics than those attending village clinics, but their overall chance of being prescribed with any type of antibiotic was lower. Overall, 87.8% of RTI patients received antibiotic prescriptions and 35.5% of these contained two or more types of antibiotics, with the most commonly prescribed being broad spectrum antibiotics levofloxacin (31.0% in township health centres and 30.9% in village clinics) and amoxicillin (25.6% in township health centres and 36.3% in village clinics). 45.8% of the 1068 RTI clinic patients reported that they had already used antibiotics in the past year, with 77.1% stating that they had obtained these from a hospital pharmacy (implying a previous clinical consultation) and 44.4% reporting that they had obtained them elsewhere. At retail pharmacies, of 2,216 observed customer purchases, 357 were observed to purchase antibiotics without prescription for any reason. Purchased medicines for RTIs at retail pharmacies included a lower proportion of antibiotics (16.9%); no customers describing UTI symptoms were observed purchasing medication from retail pharmacies.

**Table 2:** Use of antibiotics in different settings.

	Township health centres (THC)		Village Clinics (VC)		Retail pharmacies	
	N	%	N	%	N	%
*Mode of administration*						
-Oral antibiotics	382	57.2	251	55.2	122	16.9
-Intravenous antibiotics	304	45.5	187	41.1	0	0
-Oral and intravenous antibiotics	123	18.4	14	3.1	0	0
-Oral or intravenous antibiotics	563	84.3	424	93.2	122	16.9
*Number of antibiotics used in combination*						
-Zero	105	15.7	31	6.8	592	82.2
-One	339	50.8	236	51.9	122	16.9
-Two	168	25.2	151	33.2	6	0.8
-Three or more	56	8.4	37	8.1	0	0
*Top 10 most commonly provided antibiotics in THC/VC/medicine shops*						
Levofloxacin/Levofloxacin/Amoxicillin	220	32.9	158	34.7	45	39.1
Amoxicillin/Amoxicillin/Cefixime	165	24.7	155	34.1	18	15.7
Erythromycin/Cefradine/Cefradine	163	24.4	54	11.9	13	11.3
Cefuroxime/Ceftriaxone/Roxithromycin	87	13.0	57	12.5	12	10.4
Ceftazidime/penicillin/Spiramycin	41	6.1	50	11.0	10	8.7
Cefotaxime/Ampicillin/Erythromycin	24	3.6	38	8.4	5	4.4
Piperacillin/Cefuroxime/Cefaclor	22	3.3	20	4.4	3	2.6
Cefaclor/Roxithromycin/Levofloxacin	21	3.1	17	3.7	3	2.6
Aloxicillin/Clindamycin/Azithromycin	21	3.1	15	3.3	2	1.7
Cefradine/Ceftazidime/Cefalexin	19	2.8	15	3.3	2	1.7
Other	56	8.4	64	14.1	10	8.7

### Bacterial pathogens and antibiotic resistance

683 of the 1068 RTI patients seen in clinics provided sputum samples, from which bacterial isolates were obtained of *K*.*pneumoniae* (n = 71), *H*.*influenzae* (52*)*, *H*.*parainfluenzae* (28*)*, *P*.*aeruginosa* (20), *H*.*parahaemolyticus* (14), *S*.*aureus* (11), *M*.*catarrhalis* (10) *E*.*aerogenes* (8), *S*.*pneumoniae* (8), *E*.*cloacae* (6) and 41 other bacteria. 385 of RTI patients provided throat swab samples, from which bacterial isolates were obtained of *H*.*parainfluenzae* (n = 24), *K*.*pneumoniae* (14), *S*.*aureus* (7), *E*.*cloacae* (6), β-Haemolytic Streptococcus (5), *H*.*influenzae* (5), *H*.*parahaemolyticus* (3), *M*.*catarrhalis* (2), *P*.*aeruginosa* (2), *H*. *haemolyticus* (2) and 13 other bacteria. 55 patients with UTI provided mid-stream urine samples which yielded 23 strains of E.coli and 5 other bacteria.

Comparison of these results with national surveillance data (CARRS, 2018) shows that rates of antibiotic sensitivity were higher for many bacterial species collected from sputum, swab or urine specimens when compared to the published data, as shown in Table 3. Susceptibility rates of *E*.*coli* from urine specimens was higher than CARRS data for almost all antibiotics tested, with particularly large differences in resistance seen for aztreonam (86.9% vs 58.3%), ceftriaxone (91.3% vs 76%) andciprofloxacin 73.9% vs 45.3%) and and 100% for amikacin, imipenem and meropenem (Table 3). Among *K*.*pneumoniae* from the respiratory tract, >95% of isolates were sensitive to the 13 antibiotics tested, markedly more sensitive than reported through CARRS. In contrast to these two species, sensitivity rates in *P*.*aeruginosa* were broadly similar to CARRS data while for the four other species tested, numbers of individual strains were low or comparative data was lacking, making comparison difficult.

**Table 3:** Bacterial sensitivity rates using CLSI standards for Anhui Province from current study (S) and from China National Surveillance System (CARRS).

Agent	% susceptible													
	*K*.*pneumoniae*^##^ (n = 85)		*H*.*influenzae*^##^ (n = 57)		*M*.*catarrhalis*^##^ (n = 12)		*S*.*pneumoniae*^##^ (n = 8)		*S*.*aureus*^##^ (n = 18)		*E*.*coli*^#^ (n = 23)		*P*.*aeruginosa*^##^ (n = 22)	
	S	CARRS	S	CARRS	S	CARRS	S	CARRS	S	CARRS	S	CARRS	S	CARRS
penicillin	-		-		-		100	96.9	7.7	4.9	-		-	
ampicillin/amoxicillin	1.3	-	28.0	39.7	5.0	-	-		-		26.7	14.9	-	
ampicillin/sulbactam	95.8	54.9	54.9	58.1	81.8	-	-		-		46.7	47.1	-	
flucloxacillin	-		-		-				100	-	-		-	
aztreonam	100	65.5	76.0	-	80	-	-		-		86.9	58.3	77.8	72.5
cefazolin	96.2	43.4	-		-		-		-		33.3	21.7	-	
cefepime	98.7	73.0	-		100	-	-		-		91.3	76.0	88.9	85.9
cefotaxime/ceftriaxone	100	59.3	100	-	75	-	-		-		60.9	40.2	-	
ceftazidime	100	70.4	91.1	-	83.3	-	-		-		86.9	70.7	85.0	83.4
cefuroxime	96.2	51.7	73.1	65	-		100	-	-		52.6	50.6	-	
piperacillin/tazobactam	98.7	80.2	-		-		-		-		95.6	96.7	88.2	86.1
ciprofloxacin	98.8	70.7	82.4	-	83.3	-	-		94.1	-	73.9	45.3	80.0	82.0
moxifloxacin	-		-		-		100	97.2	-		-		-	
amikacin	100	84.7	-		100	-	-		-		100	96.2	89.5	93.2
gentamicin	98.8	69.8	-		91.7	-	-		100	85.9	71.4	58.6	90.0	88.7
imipenem	98.7	82.2	-		100	-	-		-		100	98.2	84.2	79.9
meropenem	100	80.0	-		100	-	100	-	-		100	99.1	94.7	82.9
tetracycline	-		73.7	-	-		-		81.2	-	-		-	
nitrofurantoin	48.1	-	-		-				100		85.7	-	-	
chloramphenicol	-		-		-		-		-		-		-	
co-trimoxazole	-		-		-		-		-		-		-	
erythromycin	-		-		-		0	4.7	64.7	38.5	-		-	
clindamycin	-		-		-		20	10.1	-		-		-	
linezolid	-		-		-		-	-	100	100	-		-	

*Key*:‘–’represents data not available

^**# #**^ represents strain isolated from sputum or throat swab samples

^**#**^ represents strain isolated from urine samples.

### Determinants of antibiotic prescribing for respiratory tract infections

Relationships between antibiotic prescribing (using clinic recruitment and patient exit survey data) and bacterial isolation and antibiotic resistance (using microbiological data) were then explored statistically. Because numbers of patients presenting to outpatient clinics with UTI symptoms were unexpectedly low (55, 4.9%), the statistical analysis in this section focuses only on RTI patients (Table 4). Multivariate logistic regression modelling (using IV, oral and IV/oral antibiotic use as the dependent variable respectively and the same set of independent variables as specified in Table 4) revealed that antibiotic prescription was: not linked with bacteria isolation and resistance detection; weakly linked with one type of symptom, i.e. sore throat (OR = 1.56, 95% CI 0.99–2.46); and strongly linked with study site (OR = 4.2, 95% CI 2.7–6.53)) and longer duration (>10 days) since infection onset (OR = 3.33, 95% CI 1.49–7.43)). Fever was found to be a predictor for prescription of intravenous antibiotics (OR = 2.87, 95% CI 1.94–4.27) and older age groups also had greater likelihood of receiving IV antibiotics (OR = 1.5, 95% CI 1.03–2.18 in 40–59 year-olds and OR = 1.63, 95% CI 1.1–2.4 in 60–89 year-olds). Overall, antibiotic prescribing was not associated with previous treatment for the current infection (OR = 1.14, 95% CI 0.68–1.9)), though exit survey data showed that a quarter (272, 25.47%) of patients reported having used antibiotics prior to visiting the health care facility. However, patients who reported having received IV antibiotics before the study consultation had a higher chance of being prescribed IV antibiotics again and were less likely to receive oral antibiotics. Although only small numbers of RTI patients had specifically asked for IV or oral antibiotics during their consultation (8.49% and 13.42% respectively), these clearly stated requests were strongly linked with prescription of intravenous (OR = 6.77, 95% CI 3.75–12.21) and oral (OR = 3.05, 95% CI 1.91–4.89) antibiotics, respectively. Additional analyses that further explore antibiotic prescribing, clinical diagnosis and bacterial detection in relation to types and numbers of antibiotics used by clinical diagnosis and pathogen identification and in relation to patient sociodemographic characteristics are reported separately [28, 29].

**Table 4:** Findings from multivariate logistic regression modeling of antibiotics use for respiratory tract infections (RTI) in clinic patients.

Independent variables	IV or oral antibiotics (n = 987)			IV antibiotics (n = 491)			Oral antibiotics (n = 633)		
	OR	95% C.I.		OR	95% C.I.		OR	95% C.I.	
		Lower	Upper		Lower	Upper		Lower	Upper
**Type of setting**									
*village clinics*	Ref.			Ref.			Ref.		
*township health centres*	3.34^*^	2.02	5.52	1.12	0.79	1.57	0.94	0.68	1.30
**Study sites**									
*site 1*	Ref.			Ref.			Ref.		
*site 2*	1.88	0.99	3.59	0.47^*^	0.30	0.73	4.20^*^	2.70	6.53
*site 3*	1.26	0.66	2.43	0.39^*^	0.25	0.62	1.97^*^	1.25	3.09
*site 4*	0.69	0.30	1.60	0.21^*^	0.10	0.44	2.36^*^	1.26	4.43
**Sex**									
*male*	Ref.			Ref.			Ref.		
*female*	0.82	0.55	1.23	1.02	0.76	1.37	0.83	0.63	1.10
**Age**									
*18–39*	Ref.			Ref.			Ref.		
*40–59*	1.71^*^	1.03	2.82	1.50^*^	1.03	2.18	0.95	0.67	1.35
*60–89*	1.96^*^	1.16	3.31	1.63^*^	1.10	2.40	0.91	0.63	1.32
**Days since onset of infection**									
*within 1 day*	Ref.			Ref.			Ref.		
*2 to 10 days*	1.92	0.91	4.05	1.84	0.88	3.85	1.60	0.81	3.18
*10 days or more*	3.33^*^	1.49	7.43	2.59^*^	1.22	5.50	1.80	0.89	3.64
*not clearly stated*	1.08	0.45	2.61	1.49	0.62	3.61	0.87	0.38	2.02
**Reported RTI symptoms**									
*Discharge*	1.57	0.59	4.18	0.71	0.39	1.30	1.94^*^	1.07	3.53
*Cough with sputum*	0.70	0.47	1.06	0.79	0.58	1.08	0.90	0.67	1.21
*Sore throat*	1.56	0.99	2.46	1.00	0.74	1.37	1.16	0.86	1.56
*Itchy throat*	1.44	0.57	3.59	0.92	0.48	1.75	1.48	0.79	2.79
*Breathing dificulty*	0.72	0.39	1.34	1.41	0.86	2.31	0.84	0.52	1.35
*Headache*	0.86	0.41	1.78	1.25	0.74	2.11	0.83	0.50	1.36
*Weakness*	1.34	0.38	4.70	2.11	0.93	4.77	0.41^*^	0.18	0.92
*Fever*	1.52	0.82	2.81	2.87^*^	1.94	4.27	0.56^*^	0.39	0.82
**Clearly stated request for IV by patient**	3.62^*^	1.26	10.37	6.77^*^	3.75	12.21	0.28^*^	0.17	0.47
**Clearly stated request for medicines by patient**	0.82	0.47	1.43	0.09^*^	0.04	0.19	3.05^*^	1.91	4.89
**IV treatment before current consultation**	0.82	0.36	1.87	2.11^*^	1.03	4.33	0.47^*^	0.23	0.95
**Medicine use before current consultation**	1.14	0.68	1.90	1.71^*^	1.16	2.52	0.70	0.49	1.02

*Note*: * represents *p* value >0.05

### Drivers of antibiotic use

#### Clinical encounters

Clinical consultations were generally short (<5 minutes) and contained minimal exchange of information; history-taking was rare. Patients typically described their symptoms briefly and throat or pulse was sometimes examined before the doctor wrote a prescription or (in township health centres where facilities are available) an order for a blood test or X-ray. Consultations took place in the presence of other patients waiting their turn, returning with test results, or waiting to receive guidance on medication use. Most over-the-counter purchases of antibiotics at retail pharmacies also involved limited consultation, with nearly half (47%) of medicines purchased without prescription being selected by customer request and only 53% decided by the pharmacy worker after symptoms were described.

**Fig 2:**
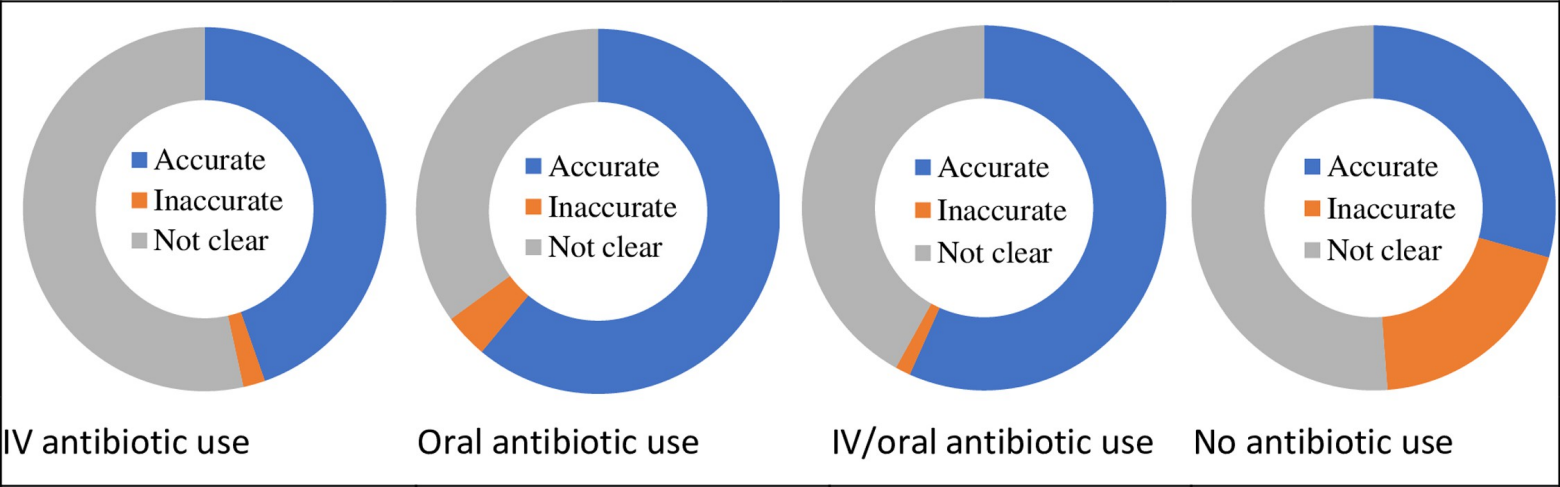
Accuracy of patient-reported antibiotics use compared with direct observations.

The brevity of clinic encounters may explain why in exit interviews, only half (56.2%) of clinic patients who had been *observed* to receive antibiotic prescriptions *reported* that their prescription included antibiotics, while a similar proportion of antibiotic-purchasing pharmacy customers (46.98%) said the medicine they had just bought contained antibiotics (Fig 2). A higher proportion of clinic patients prescribed oral antibiotics confirmed that their prescriptions contained antibiotics (61%) than those prescribed antibiotics for intravenous administration (47%), probably because IV was described generically by reference to the mode of administration rather than its contents.

#### Doctors’ and patients’ views

Among the 30 doctors interviewed from all participating clinical facilities, over half indicated that diagnostic uncertainty drives antibiotic prescribing. The perceived need to provide effective treatment and ensure patient recovery frequently resulted in combined prescribing (orally or intravenously) of an antibiotic and a Traditional Chinese Medicine preparation regarded as anti-viral:

“As a doctor, I think that if you aren’t sure then all you can do is choose to use both types together. Because if you can’t confirm what it is and if you only use, say, antibiotics (*kang sheng su*), anti-inflammatories *(xiao yan)*, when in fact it is a viral infection, then you won’t have good results. So all you can do is use the two together in combination” (2-1-20180131).

Although those interviewed were aware that unnecessary antibiotic use contributes to antibiotic resistance, without access to microbiological support, uncertainty regarding type of bacterial infection produced a preference for broad-spectrum and combination antibiotics:

“…if you aren’t certain what type of microbial infection (*jun gan ran*) it is, then generally you would use broad-spectrum antibiotics (*kang sheng su*)… sometimes combining two or three is definitely a bit better. Why do I say a bit better? It’s because you aren’t certain which type of antibiotic (*kang sheng su*) is better for the infection” (2-1-20180201).

Such prescribing practices were described as protecting patient safety (including preventing risk of secondary infection), satisfying perceived patient demand for rapidly effective treatment, and safeguarding doctors against the potential reputational risk of failing to treat successfully.

”Now, the patients are anxious to achieve quick success and get instant benefits, if you do not use antibiotics, if the effect is not good, the patient will bring you trouble” [1–2–20170626].

Some doctors linked this concern, along with the difficulty of refusing to prescribe antibiotics, to the low social status of both rural doctors and the poor populations they serve:

“The social status of the base-level clinicians is low. […] If the medical treatment works to [the patient’s] recovery, it is your job done; but if it doesn’t work, you are to be blamed […]. Many families here are the ‘left-behind’ households, with young parents working as migrants away in the city [while grandparents look after the children]. Clinicians end up bearing the brunt if any social problems arise. (2-1-20180115).

Some doctors also highlighted the need to earn income for themselves and their health facility as driving their prescribing practices. Following the successful implementation of the Essential Medicines Policy (2009), health facilities cannot mark up the costs of oral medicines, but provision of clinical services including tests and parenteral drug administration are economically important: “But now the hospital[s], in order to raise income, they give patient[s] infusion treatment easily” [2–1–20180115]; “If you give the patient infusion [IV antibiotics], the effect is faster, it will attract more patients, the income will be better.”[1–2–20170625] The influence of social position and economic considerations on antibiotic prescribing among village doctors is further explored in a separate publication [30].

Another driver of frequent antibiotic use is the characterisation of antibiotics as ‘anti-inflammation medicine’. As described elsewhere in greater depth, [31] biomedical and local understandings of infection and inflammation are elided in contemporary clinical practice in these settings. Most doctors used either the colloquial descriptor (*xiaoyan yao*, literally ‘anti-inflammation medicine’) or the specific drug name (e.g. amoxicillin) rather than the biomedical term for ‘antibiotic’ in patient consultations, explaining this as necessary for communication:

“The common people won’t understand if I say ‘antibiotics’ (*kang sheng su*). They do not know ‘antibiotics’ (*kang sheng su*), they only know ‘anti-inflammation medicine’ (*xiaoyan yao*)” (2-1-20180115).

“I use the drug name of antibiotics to communicate with patient”; […] If you say antibiotic or antibacterial drug, they can’t understand” [1–2–20170625]

Interviews with patients, however, overwhelmingly confirmed that common symptoms of infection including sore throat, coughing, fever, redness and swelling are recognised as indicating the presence of inflammation. Since antibiotics are ‘anti-inflammation medicine’ (*xiaoyan yao*), patients logically regard antibiotics as appropriate treatment and frequently referred to encounters with healthcare personnel as a source of this knowledge:

*Interviewer*: How do you know, that when you have a cold or a cough, that you need to take anti-inflammatories?

*Interviewee*: Everyone knows it. When you go to the drug store, drug salespersons or pharmacists will tell you this; if you go to clinics, doctors will tell you this.

*Interviewer*: Tell you what? That you need anti-inflammatories?

*Interviewee*: Yes. And they will treat you with anti-inflammatories. *[1–1–20170604–1]*

### Accuracy of electronic records to monitor antibiotic prescribing

**Fig 3:**
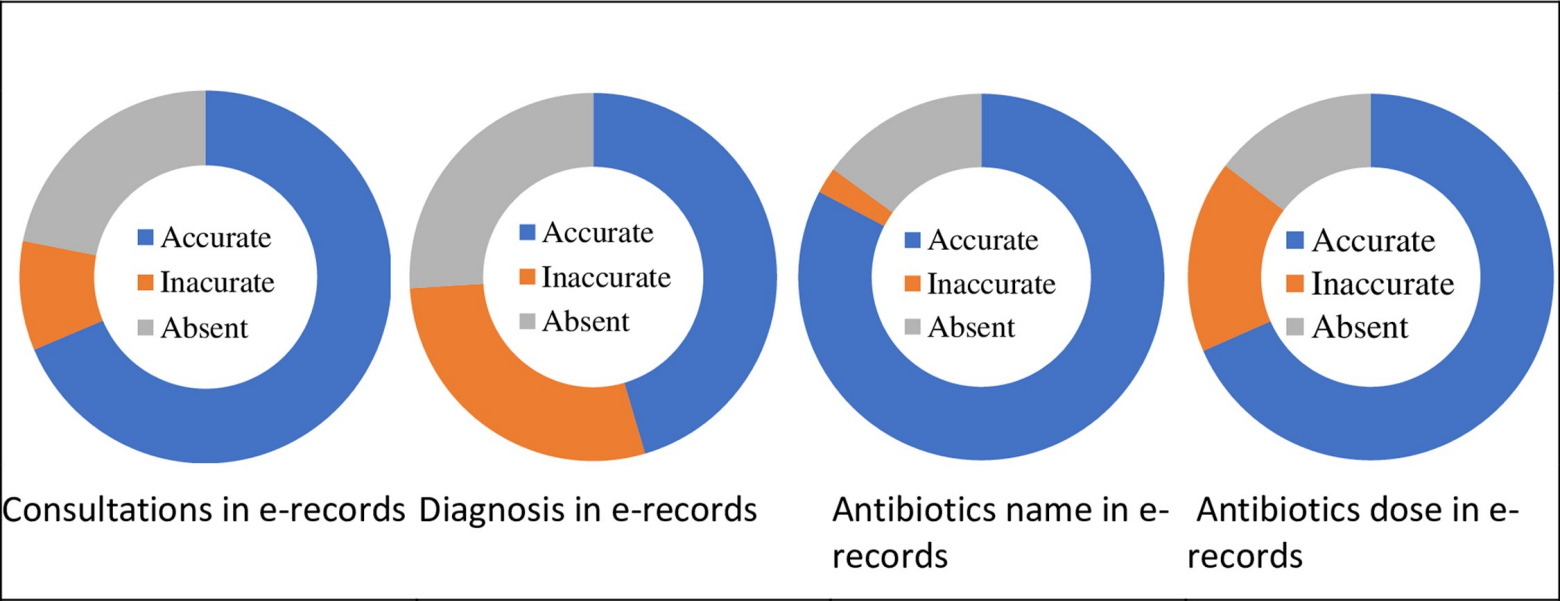
Accuracy of electronic records compared with direct observations.

There is considerable between-site variation in the completeness of e-records (0% to 98.7% of clinic consultations) and between physicians, as well as variability in when records are completed (from within-consultation, through days or weeks later, to annually when prescription audit requires paper records to be entered electronically). E-records are used much less frequently in village clinics than in township health centres, due to low computer literacy levels and poor connectivity. E-records were created only for individuals with health insurance. E-records were found for 781 (75.7%) of the 1030 patients who had been observed in these clinics; the remaining 25% either did not have e-records or were not retrievable from patient identifiers (name, date of birth). E-record accuracy was better in relation to antibiotics (82.8% of e-records accurately recorded what was prescribed in clinic) than for diagnosis (45.0% accuracy) and symptoms (1.1% accuracy) (Fig 3). E-records recorded antibiotic names with a high degree of accuracy (647/781, 82.8%) and the correct doses were recorded in in 534/781 (68.4%). E-records from township health centres (n = 636) recorded the administration route for antibiotics (IV/IM/oral) but not those from village clinics. Across all clinic consultations a total of 51 types of antibiotic were prescribed, while retrieved e-records documented only 38 different antibiotic types. Discrepancies were greatest for amoxicillin capsules, representing 15.6% of all antibiotic prescriptions recorded during observation but only 6.9% of antibiotic e-prescriptions; ceftriaxone sodium for injection, prescribed in 5.9% of observed consultations but 3.8% of antibiotic e-prescriptions; and levofloxacin lactate sodium for injection, which constituted 10.6% of observed antibiotic prescriptions and 8.2% of e-prescriptions.

## Discussion

This study confirmed that rates of antibiotic prescribing and consumption remain high in rural eastern China. The Chinese Medical Association’s *Guidelines for Rational Use of Drugs in Primary Care for Acute Upper Respiratory Tract Infection* (2020) state that bacterial infection only accounts for 20–30% of diagnosed patients [32]. The Chinese Medical Association has also published guidelines on primary diagnosis and treatment of acute tracheobronchitis, COPD and cough in general practice, [33–35] but according to government policy as stated in *Assessment Indicators and Requirements for clinical application management of Antibacterial Drugs*, [36] no more than 20% of prescriptions in outpatient clinics of secondary medical institutions in China should be antibiotics. Our study found that the rate of antibiotic prescribing in primary medical institutions is far higher than this.

The results from different methodological approaches converge to enhance their validity; thus our exit survey finding that roughly a quarter of those attending low level health facilities for common RTIs (those most frequently diagnosed in primary care including bronchitis/tracheitis, upper respiratory tract infection, pharyngitis, common cold, pneumonia/bronchopneumonia and tonsillitis) or UTIs had received antibiotics for previous illness episodes in the past year, and that two-thirds of these were from a hospital pharmacy, concords with our observational data on antibiotic prescribing practices in these facilities. The findings that a further third of clinic patients had obtained antibiotics outside hospital pharmacies, and that nearly 90% of retail pharmacy customers had not previously sought treatment elsewhere, further suggest that over-the-counter purchasing of antibiotics as a first resort for everyday health problems is not uncommon, and this is consistent with previous studies that provide evidence for purchasing of antibiotics without prescription in various settings and population groups in China despite ongoing government efforts to restrict this [25]. From the perspective of patients, frequent antibiotic prescribing for common RTIs in clinics reinforces perceptions that antibiotics are appropriate for common symptoms, indirectly validating their direct purchasing of antibiotics to self-treat such symptoms.

Analysis of clinic patient presenting symptoms in relation to prescriptions suggests that empirical prescribing of antibiotics is particularly associated with presence of sore throat and, in the case of intravenous antibiotics, fever. Our findings further indicate that these symptoms are viewed by both patients and doctors as indicating the presence of ‘inflammation’, for which antibiotics (as ‘anti-inflammation medicine’) are considered appropriate. The lack of an association between presence of bacterial pathogens and prescription of antibiotics reinforces the difficulty of determining clinical need for antibiotics in the absence of microbiological diagnostic tests. Interviews with doctors confirmed that while they know these conditions may not all be bacterial, they are concerned about the possibility of secondary infections and, in these settings where patients have low incomes and can rarely afford time off work and domestic duties, they regard patient recovery as most reliably ensured by prescribing broad spectrum antibiotics, in combination with an anti-viral traditional Chinese medicine to cover all possible causes. Previous studies have identified uncertainty about treatment and lower levels of professional training as associated with higher levels of antibiotic prescribing [25]. Suboptimal antibiotic prescribing is exacerbated by short consultations with limited history-taking and non-disclosure of prior antibiotic consumption by patients. Consequently, doctors are frequently unaware of antibiotics already taken by consulting patients, and patients are not usually told exactly what medicines they have been prescribed. This increases the potential for development of resistance to the most commonly used classes of antibiotic through repeated use (including self-medication), reflected in resistance rates of 80% to amoxicillin for *E*.*coli* and 54% for *H*.*influenzae*, for example. Interventions to optimise prescribing practices among rural doctors need to address patient understandings of antibiotics and, given the availability of over-the-counter antibiotics, be accompanied by measures to involve retail pharmacies in antibiotic stewardship. However, analysis of our clinic observations contradicts assertions made by some doctors in our interviews that their antibiotic prescribing is a response to patient demand. While we found that direct patient requests for oral and especially IV antibiotics were strongly linked with antibiotic prescribing, such requests were only observed in a small minority of clinical consultations (13.42% and 8.49% for oral and IV respectively), and doctors were often seen to prescribe antibiotics spontaneously. This suggests that, as reported elsewhere, antibiotic prescribing is more closely associated with doctors’ assumptions about what patients want than with patients’ actual expectations [37].

The implications of our findings regarding the use of intravenous antibiotics in outpatients for antibiotic stewardship warrants particular attention. Our interview and exit survey data suggest that patients value IV infusion as an effective medical intervention *per se* that works faster than oral medication. Less than half (47%) of those prescribed intravenous antibiotics knew what medicines they were receiving, while doctors explained that providing this ‘service’ helps to generate income for their health care facility. In the short term, doctors could be advised to reduce the use of antibiotics in infusions (which usually also contain traditional Chinese medicine anti-viral formulations) while in the longer term, patient-level measures to increase awareness of the risks of IV antibiotic administration and system-level interventions to modify economic incentives for prescribing infusions could be considered.

Importantly, our microbiological data demonstrate that despite high rates of antibiotic prescribing and dispensing, resistance rates are low in comparison with published surveillance data. The participating laboratory in our study is a member unit of CARRS national surveillance system using similar tests and standards so our drug sensitivity results are comparable to CARRS. The lower resistance rates found in our study are therefore probably due to the presence of hospital-acquired resistant infections and referred patients who have already acquired resistance being overrepresented in the data from urban tertiary hospitals which participate in the CARRS national surveillance system.

Our study was limited to eight health facilities in rural areas of one Province and therefore may have limited generalisability for rural China as a whole, although study sites were randomly sampled within the parameters of our selection criteria. Our selection of study sites was also geographically limited by the need to transport samples to our reference laboratory in Hefei (the provincial capital) by public transportation within four hours; however, this microbiological study has nonetheless demonstrated that it is feasible to assess antibiotic resistance rates in bacterial pathogens from patients in rural China. A further limitation of our study was very limited recruitment of UTI patients, who unexpectedly did not often present at these frontline health facilities. Possible reasons for this may include self-referral to more distant higher-level facilities, perhaps because of stigma arising from a perceived association between UTI symptoms and those of sexually transmitted infections (which in China’s national health system require mandatory referral to a specialist facility), resulting in patients avoiding seeking treatment at facilities where they are known, or low prevalence of UTI in this population, but this issue requires further investigation.

Our record review study has important implications for use of e-records for ABR surveillance and monitoring effectiveness of antibiotic stewardship interventions. E-records facilitate reimbursement from state medical insurance and accordingly, are created using a patient’s medical insurance number; where the patient has no insurance ID or has exceeded the individual annual reimbursement allowance for state-funded care, doctors may create e-record(s) using the ID of one or more family members to help cover costs. The use of e-records primarily for the purpose of insurance reimbursement rather than for patient care accounts for the high degree of accuracy and completeness of antibiotic prescriptions (82.8%) and doses (68.4%) as compared with that for symptoms (1.1%) and diagnoses (45%). These findings are explored in greater detail in a separate paper [38]. Combining direct observation with epidemiological methods demonstrates that the widespread use of secondary patient data to monitor trends in antibiotic prescribing may conceal substantial biases. Whilst e-records are potentially a valuable source of epidemiological intelligence, they should be interpreted with caution, particularly where electronic reporting systems are not used universally or integrated across the entire health care system.

## Conclusion

In the first study from China on antibiotic resistance in rural communities, we demonstrated the feasibility of obtaining and culturing microbiological samples to ascertain community prevalence of resistance in rural settings. Our findings show that while antibiotic prescribing rates were very high for common respiratory tract infections, prevalence of antibiotic resistance was consistently lower than that reported through national surveillance. This implies that population prevalence in China is probably lower than previously assumed, consistent with antibiotic resistance prevalence rates being elevated in the large urban tertiary facilities that participate in the national surveillance system.

We have also identified diagnostic uncertainty, limited doctor-patient communication, perception of antibiotics as anti-inflammatory, and (for intravenous antibiotics) financial incentives as key drivers of antibiotic use. While self-medication with antibiotics was not unusual and a quarter of recruited patients had consumed antibiotics prior to their first visit to the recruiting clinic, only a small minority of patients directly requested antibiotics in clinical consultations; and only half of clinic attenders and pharmacy customers were able to verify whether their prescription or purchase included antibiotics. This evidence suggests that unwarranted antibiotic prescribing in clinical settings does not result principally from specific patient demand. Measures to optimise antibiotic prescribing and consumption in rural settings need to address health system drivers, including insurance reimbursement mechanisms, remuneration for clinical services that indirectly incentivises intravenous administration of antibiotics, and socioeconomic position of rural doctors; improved diagnostic support, tailored clinical guidelines and in-service training for rural doctors to manage clinical uncertainty and patient consultations; better regulation of retail pharmacies and supply chains; and public engagement to address the perception of antibiotics as inflammation-reducing and the valorisation of intravenous administration of medicines.

Comparison of electronic patient records with directly observed doctor-patient consultations shows that e-records are often incomplete and inaccurate, particularly in village health facilities where some doctors have limited expertise in the use of computerised systems. E-records’ variable accuracy and completeness have important implications for monitoring and surveillance of antibiotic prescribing. Analyses of the effectiveness of policies aiming to change antibiotic prescribing practices that are based on such data should be interpreted with caution.

Our study demonstrates the value of a conceptually unified cross-disciplinary approach, in which different methodological approaches provide insights beyond those which can be derived from solely epidemiological, microbiological, or social science study designs. The use of qualitative interviews provided insights into the reasons for frequent antibiotic prescribing and retail purchasing, while the use of direct observation drawing on ethnographic approaches made visible structured differences between recorded and actual diagnoses and prescriptions, and between local and national patterns and prevalence of antibiotic resistance. Combining qualitative, observational and survey methodologies with microbiological and epidemiological approaches to the study of antibiotic resistance from research design stage substantially increases study robustness and the validity of research findings for use in the development of future antibiotic stewardship interventions.
